# Complete Genome Sequence of Bacteriophage Fizzles, Isolated from Microbacterium foliorum

**DOI:** 10.1128/MRA.01077-21

**Published:** 2022-01-06

**Authors:** Skyler Adams, Gabrielle Spotz, Riley Babcock, Chloe Butler, Samantha Conger, Madison Crew, Stephanie Garcia, Joanna Gonzalez, Jocelyn Hodges, Alondra Martinez, Samuel Munoz, Chloe O’Grady, Abigail Quirl, Kristin Sefcik, Tanner Taylor, Gustavo Vazquez, Faith Cox, Dustin Edwards

**Affiliations:** a Department of Biological Sciences, Tarleton State University, Stephenville, Texas, USA; Queens College CUNY

## Abstract

Microbacteriophage Fizzles has a 62,078-bp linear double-stranded DNA genome sequence, predicted to contain 104 protein-coding genes. Fizzles is a *Siphoviridae* actinobacteriophage isolated from an ant hill soil sample collected in Stephenville, TX. Microbacteriophage Fizzles has >83.6% nucleotide identity with microbacteriophages Squash and Nike.

## ANNOUNCEMENT

**FIG 1:**
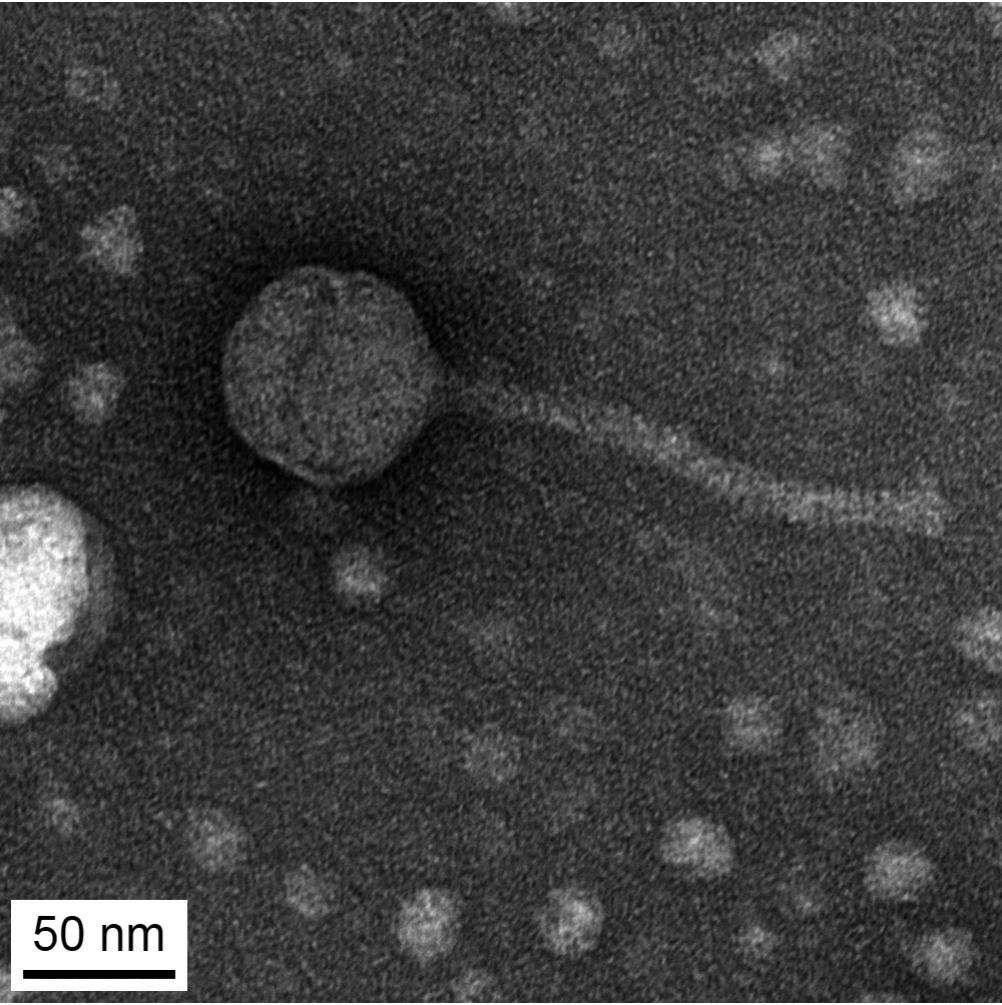
Transmission electron microscopy (TEM) of microbacteriophage Fizzles. High-titer lysate was placed on a carbon type-B 300-mesh grid, stained with 1% uranyl acetate, and imaged using an FEI Tecnai G^2^ Spirit BioTwin instrument (NL1.160G). TEM micrographs of negatively stained Fizzles particles show a tail length of 160 nm and an icosahedral capsid 65 nm in diameter.

To further understand bacteriophage genomic diversity as part of the Howard Hughes Medical Institute Science Education Alliance Phage Hunters Advancing Genomic and Evolutionary Science program (1), we report the genome sequence of microbacteriophage Fizzles (2). Fizzles was extracted from dry soil collected from an ant bed in Stephenville, TX, USA (global positioning system [GPS] coordinates, 32.2197N, 98.1989W). The soil samples were washed with peptone-yeast extract-calcium (PYCa) liquid medium, and bacteriophages were extracted through a 0.22-μm filter. The filtered medium was mixed with soft agar containing Microbacterium foliorum strain SEA B-24224, overlaid on PYCa agar, and incubated at 29°C for 48 h. Bacteriophage replication produced small, lytic plaques. Fizzles was isolated by two rounds of picking a single, well-separated plaque, followed by diluting the bacteriophage sample in a 10-fold dilution series and plating with *M. foliorum*. Negative-staining transmission electron microscopy showed *Siphoviridae* morphology, with a tail length of 160 nm and an isometric capsid 65 nm in diameter (Fig. 1), as measured using ImageJ v1.53m (3).

High-titer lysate was produced by flooding “webbed” plates, as described in the *Phage Discovery Guide* (4). Genomic DNA was extracted from high-titer lysate using a modified zinc chloride precipitation method (4, 5). Genomic sequencing libraries were prepared using the NEBNext Ultra II kit (New England Biolabs, Ipswich, MA) and sequenced using an Illumina MiSeq instrument at the Pittsburgh Bacteriophage Institute (Pittsburgh, PA). Sequencing was performed to 2,719-fold coverage from 1,194,881 total reads of 150-bp read length. Assembly to produce a single-bacteriophage contig was performed using Newbler v2.9 with default settings, and Consed v29.0 was used for assembly of a single bacteriophage contig to ensure performance for quality control (6, 7). Fizzles has a double-stranded linear DNA genome 62,078 bp long, with 181-bp direct terminal repeats and a 68.2% G+C content. Whole-genome nucleotide alignment using BLASTn (https://blast.ncbi.nlm.nih.gov/) (8) showed that Fizzles has a nucleotide sequence identity of >83.6% with *Microbacterium* phages Squash (GenBank accession number MH153813) and Nike (MT114166).

Initial auto-annotation was performed using GLIMMER v3.02 (9) and GeneMark v2.5p (10, 11), and the annotation was manually refined using Phamerator (http://phamerator.org/), DNA Master v5.23.2 (http://phagesdb.org/DNAMaster/), and PECAAN. No tRNA genes were identified using Aragorn v1.2.38 (12) and tRNAscan-SE v2.0 (13). Putative functions for 72 of 104 predicted protein-coding genes were assigned using NCBI BLASTp (8) and HHpred v3.0beta (14, 15). All tools were run with default parameters. Start codon usage was 77.7% for AUG, 21.4% for GUG, and 0.97% for UUG. Genes were transcribed rightward (48.1% of genome) and leftward (51.9% of genome) and encode structural proteins, histidine triad nucleotide-binding protein, hydrolase, MazG-like nucleotide pyrophosphohydrolase, HNH endonuclease, RuvC-like resolvase, DNA primase/helicase, RecA-like DNA recombinase, nucleotide pyrophosphohydrolase, and DnaJ-like chaperonin.

### Data availability.

The genome sequence for microbacteriophage Fizzles is available at GenBank under accession number MW924638. The raw reads are available under SRA accession number SRX11067172.
